# (4-Hydr­oxy-3-nitro­benz­yl)methyl­ammonium chloride

**DOI:** 10.1107/S160053680800473X

**Published:** 2008-02-27

**Authors:** Grant A. Boyle, Hendrik G. Kruger, Glenn E. M. Maguire, Jason Paraskevopoulos

**Affiliations:** a School of Chemistry, University of KwaZulu-Natal, Durban 4000, South Africa

## Abstract

The title compound, C_8_H_11_N_2_O_3_^+^·Cl^−^, was synthesized as an inter­mediate in the development of a new sugar sensor. The structure displays N—H⋯Cl and O—H⋯O hydrogen bonding, as well as weak O—H⋯Cl inter­actions and π–π stacking (3.298 Å). There are two formula units in the asymmetric unit.

## Related literature

For related literature, see: James *et al.* (1995 ▶).
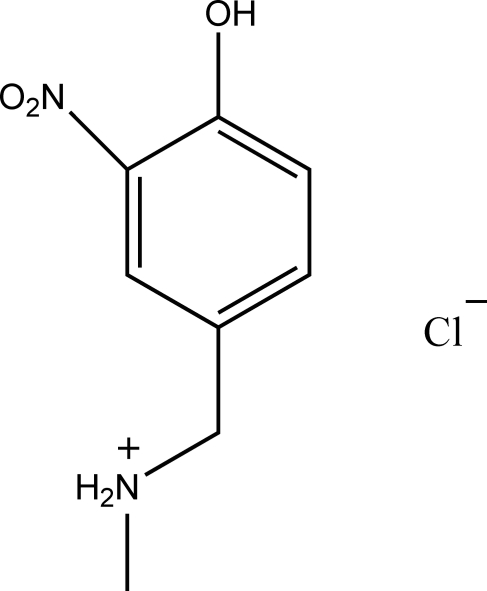


## Experimental

### 

#### Crystal data

C_8_H_11_N_2_O_3_^+^·Cl^−^
*M* *_r_* = 218.64
Triclinic, 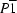

*a* = 7.7650 (2) Å
*b* = 10.5922 (3) Å
*c* = 13.5987 (4) Å
α = 70.262 (1)°
β = 78.368 (1)°
γ = 76.459 (1)°
*V* = 1014.27 (5) Å^3^
*Z* = 4
Mo *K*α radiation
μ = 0.36 mm^−1^
*T* = 173 (2) K
0.48 × 0.39 × 0.36 mm

#### Data collection

Bruker APEXII CCD area-detector diffractometer
Absorption correction: none
11798 measured reflections
4901 independent reflections
4057 reflections with *I* > 2σ(*I*)
*R*_int_ = 0.034

#### Refinement

*R*[*F* ^2^ > 2σ(*F* ^2^)] = 0.033
*wR*(*F* ^2^) = 0.094
*S* = 1.06
4901 reflections
257 parameters
H-atom parameters constrained
Δρ_max_ = 0.24 e Å^−3^
Δρ_min_ = −0.31 e Å^−3^

### 

Data collection: *APEX2* (Bruker, 2005 ▶); cell refinement: *SAINT-Plus* (Bruker, 1999 ▶); data reduction: *SAINT-Plus*; program(s) used to solve structure: *SHELXTL* (Sheldrick, 2008 ▶); program(s) used to refine structure: *SHELXTL*; molecular graphics: *Mercury* (Macrae *et al.*, 2006 ▶) and *WinGX* (Farrugia, 1999 ▶); software used to prepare material for publication: *SHELXTL* and *PLATON* (Spek, 2003 ▶).

## Supplementary Material

Crystal structure: contains datablocks I, global. DOI: 10.1107/S160053680800473X/hg2362sup1.cif

Structure factors: contains datablocks I. DOI: 10.1107/S160053680800473X/hg2362Isup2.hkl

Additional supplementary materials:  crystallographic information; 3D view; checkCIF report

## Figures and Tables

**Table 1:** Hydrogen-bond geometry (Å, °)

*D*—H⋯*A*	*D*—H	H⋯*A*	*D*⋯*A*	*D*—H⋯*A*
N2*A*—H2*A*⋯Cl1*A*	0.92	2.23	3.1301 (11)	167
N2*A*—H2*B*⋯Cl1*B*	0.92	2.18	3.0898 (11)	173
O1*A*—H1*A*⋯O2*A*	0.84	1.89	2.5917 (14)	140
O1*A*—H1*A*⋯Cl1*B*^i^	0.84	2.87	3.3918 (10)	122
N2*B*—H2*C*⋯Cl1*A*^ii^	0.92	2.17	3.0775 (11)	168
N2*B*—H2*D*⋯Cl1*B*^iii^	0.92	2.26	3.1671 (10)	168
O1*B*—H1*B*⋯O2*B*	0.84	1.88	2.5860 (14)	141

Symmetry codes: (i) 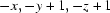; (ii) 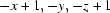; (iii) 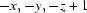.

**Fig. 1.**
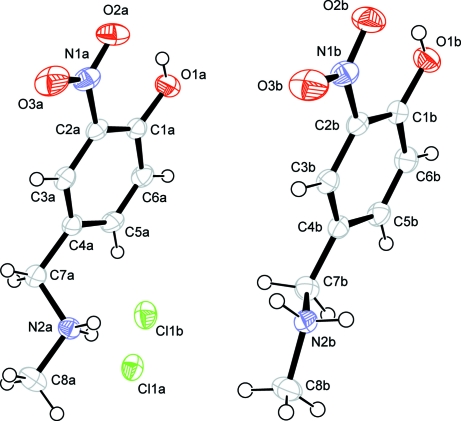
The asymmetric unit showing ellipsoids at the 50% probability level and the numbering scheme employed.

**Fig. 2.**
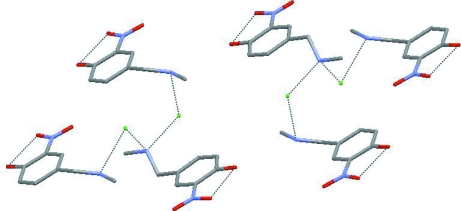
Diagram of the inter- and intramolecular hydrogen bonding. Hydrogen atoms have been omitted for clarity.

**Fig. 3.**
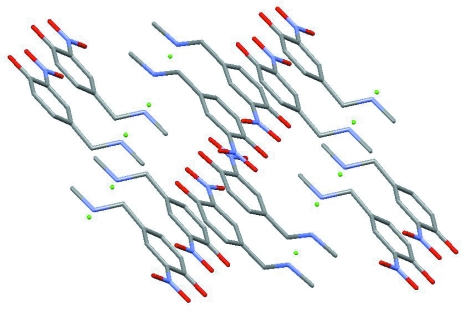
Depiction of the packing. Hydrogen atoms have been omitted for clarity.

## supplementary crystallographic information

### Comment

The title compound, (I), was synthesized as an intermediate in the development of a new sugar sensor (James *et al.*, 1995). The compound itself is also novel and is being reported for the first time.

The structure consists of two molecules in the asymmetric unit (Figure 1). The cation consists of a planar nitro phenol ring with a methylaminomethyl group in the *para* position with respect to the hydroxy group (O1) on the ring. The methylammonium groups attached to the methylene carbon (C7) deviate from the plane of the ring with a torsion angle of -121.52 (13)° for C3A—C4A—C7A—N2A and -46.81 (16)° for C3B—C4B—C7B—N2B.

The structure exhibits both intermolecular (N1—H1···Cl) and intramolecular (O1—H1···O2) hydrogen bonding interactions (Table 1, Figure 2). The chloride ions act as hydrogen bond acceptors between adjacent molecules. Weak interactions are also observed between O1—H1···Cl1. These interactions, with a bond length of 2.87Å (O1A—H1A···Cl1B^i^), are more likely weak Van der Waals interactions rather than true hydrogen bonds. See Table 1 for a full list of all hydrogen bond interactions. An interdigitated, layered structure is observed with the aromatic groups π–π stacking above each other and the methylaminomethyl group interacting with the chloride ions in hydrogen bonded layers (Figure 3).

### Experimental

4-Chloromethyl-2-nitrophenol, 3.8 g (20 mmol), was dissolved in DMF (30 ml). To this was added triethylamine (3 ml) followed by 40% methylamine in H_2_O (5 ml, 58 mmol). The reaction was heated to 333 K and left to stir overnight. The solvent was removed under vacuum to afford an orange solid, which was recrystallized from methanol at room temperature. Yield 3.49 g (80%). Decomposition point 373–375 K.

^1^H-NMR (400 MHz, D_2_O): p.p.m. = 0.00 (TMS), 2.62 (s, 3H, CH_3_), 4.12 (s, 2H, CH_2_), 7.14 (d, J = 8.5 Hz, 1H, H5), 7.60 (d, J = 8.5 Hz, 1H, H6), 8.13 (s, 1H, H3).^13^C-NMR(100 MHz, D_2_O): p.p.m. = 0.00 (TMS), 32.58 (CH_3_), 51.50 (CH_2_),121.30 (C6), 123.50 (C4), 127.75 (C3), 136.86 (C2), 139.04 (C5), 154.76 (C1).

### Refinement

Hydrogen atoms were located in the difference map then positioned geometrically, and allowed to ride on their respective parent atoms, with bond lengths of 0.99Å (CH_2_), 0.98Å (CH_3_), 0.95Å (CH), 0.98Å (NH_2_) or 0.84Å (OH). Isotropic displacement parameters for these atoms were set equal to 1.2 (CH_2_, CH and NH_2_), or 1.5 (CH_3_ and OH) times *U_eq_* of the parent atom.

### Crystal data

**Table:**

C_8_H_11_N_2_O_3_^+^·Cl^–^	*Z* = 4
*M**_r_* = 218.64	*F*_000_ = 456
Triclinic, *P*1	*D*_x_ = 1.432 Mg m^−^^3^*D*_m_ = 1.432 Mg m^−^^3^*D*_m_ measured by not measured
Hall symbol: -P 1	Mo *K*α radiation λ = 0.71073 Å
*a* = 7.7650 (2) Å	Cell parameters from 6008 reflections
*b* = 10.5922 (3) Å	θ = 4.6–28.4º
*c* = 13.5987 (4) Å	µ = 0.36 mm^−^^1^
α = 70.262 (1)º	*T* = 173 (2) K
β = 78.368 (1)º	Block, orange
γ = 76.459 (1)º	0.48 × 0.39 × 0.36 mm
*V* = 1014.27 (5) Å^3^	

### Data collection

**Table:**

Bruker SMART CCD area-detector diffractometer	4057 reflections with *I* > 2σ(*I*)
Radiation source: fine-focus sealed tube	*R*_int_ = 0.034
Monochromator: graphite	θ_max_ = 28.0º
*T* = 173(2) K	θ_min_ = 1.6º
φ and ω scans	*h* = −10→10
Absorption correction: none	*k* = −13→13
11798 measured reflections	*l* = −17→16
4901 independent reflections	

### Refinement

**Table:**

Refinement on *F*^2^	Secondary atom site location: difference Fourier map
Least-squares matrix: full	Hydrogen site location: inferred from neighbouring sites
*R*[*F*^2^ > 2σ(*F*^2^)] = 0.033	H-atom parameters constrained
*wR*(*F*^2^) = 0.094	*w* = 1/[σ^2^(*F*_o_^2^) + (0.0542*P*)^2^ + 0.0174*P*] where *P* = (*F*_o_^2^ + 2*F*_c_^2^)/3
*S* = 1.06	(Δ/σ)_max_ < 0.001
4901 reflections	Δρ_max_ = 0.24 e Å^−^^3^
257 parameters	Δρ_min_ = −0.31 e Å^−^^3^
Primary atom site location: structure-invariant direct methods	Extinction correction: none

### Special details

**Table:**

Geometry. All e.s.d.'s (except the e.s.d. in the dihedral angle between two l.s. planes) are estimated using the full covariance matrix. The cell e.s.d.'s are taken into account individually in the estimation of e.s.d.'s in distances, angles and torsion angles; correlations between e.s.d.'s in cell parameters are only used when they are defined by crystal symmetry. An approximate (isotropic) treatment of cell e.s.d.'s is used for estimating e.s.d.'s involving l.s. planes.
Refinement. Refinement of *F*^2^ against ALL reflections. The weighted *R*-factor *wR* and goodness of fit *S* are based on *F*^2^, conventional *R*-factors *R* are based on *F*, with *F* set to zero for negative *F*^2^. The threshold expression of *F*^2^ > σ(*F*^2^) is used only for calculating *R*-factors(gt) *etc*. and is not relevant to the choice of reflections for refinement. *R*-factors based on *F*^2^ are statistically about twice as large as those based on *F*, and *R*- factors based on ALL data will be even larger.

### Fractional atomic coordinates and isotropic or equivalent isotropic displacement parameters (Å^2^)

**Table:**

	*x*	*y*	*z*	*U*_iso_*/*U*_eq_	
C1A	0.07924 (17)	0.64421 (12)	0.39067 (10)	0.0280 (3)	
C2A	−0.05629 (16)	0.60295 (12)	0.36161 (10)	0.0266 (3)	
C3A	−0.02230 (17)	0.54096 (12)	0.28266 (10)	0.0277 (3)	
H3A	−0.1168	0.5129	0.2652	0.033*	
C4A	0.14689 (17)	0.52009 (12)	0.22990 (10)	0.0272 (3)	
C5A	0.28403 (17)	0.55999 (13)	0.25897 (11)	0.0311 (3)	
H5A	0.4022	0.5452	0.2238	0.037*	
C6A	0.25051 (17)	0.61995 (13)	0.33725 (11)	0.0318 (3)	
H6A	0.3462	0.6456	0.3556	0.038*	
C7A	0.17964 (18)	0.46001 (13)	0.14103 (11)	0.0328 (3)	
H7A	0.0658	0.4412	0.1318	0.039*	
H7B	0.2224	0.5269	0.0749	0.039*	
C8A	0.3333 (2)	0.26316 (15)	0.07972 (12)	0.0408 (3)	
H8A	0.2200	0.2359	0.0819	0.061*	
H8B	0.4277	0.1822	0.0934	0.061*	
H8C	0.3646	0.3263	0.0100	0.061*	
N1A	−0.23762 (14)	0.62436 (11)	0.41284 (10)	0.0339 (3)	
N2A	0.31439 (14)	0.33107 (10)	0.16086 (8)	0.0270 (2)	
H2A	0.4233	0.3503	0.1621	0.032*	
H2B	0.2807	0.2726	0.2259	0.032*	
O1A	0.06011 (13)	0.70500 (10)	0.46491 (8)	0.0388 (2)	
H1A	−0.0465	0.7123	0.4939	0.058*	
O2A	−0.27172 (14)	0.68749 (10)	0.47848 (9)	0.0457 (3)	
O3A	−0.35065 (13)	0.58060 (12)	0.39075 (10)	0.0518 (3)	
C1B	0.08090 (17)	0.14787 (12)	0.89457 (10)	0.0271 (3)	
C2B	−0.03119 (15)	0.10203 (12)	0.85044 (10)	0.0248 (3)	
C3B	0.03195 (16)	0.04521 (12)	0.76886 (10)	0.0256 (3)	
H3B	−0.0483	0.0164	0.7401	0.031*	
C4B	0.21073 (16)	0.03066 (12)	0.72966 (10)	0.0251 (3)	
C5B	0.32453 (17)	0.07415 (13)	0.77435 (11)	0.0294 (3)	
H5B	0.4486	0.0636	0.7488	0.035*	
C6B	0.26146 (17)	0.13157 (13)	0.85399 (11)	0.0317 (3)	
H6B	0.3423	0.1608	0.8821	0.038*	
C7B	0.28683 (17)	−0.02588 (12)	0.63923 (10)	0.0285 (3)	
H7C	0.2538	0.0440	0.5729	0.034*	
H7D	0.4189	−0.0455	0.6340	0.034*	
C8B	0.3304 (2)	−0.22269 (14)	0.57491 (11)	0.0383 (3)	
H8D	0.3227	−0.1618	0.5028	0.057*	
H8E	0.2844	−0.3051	0.5841	0.057*	
H8F	0.4554	−0.2478	0.5879	0.057*	
N1B	−0.22054 (14)	0.11494 (11)	0.88843 (9)	0.0311 (3)	
N2B	0.22290 (13)	−0.15229 (10)	0.65039 (9)	0.0260 (2)	
H2C	0.2293	−0.2100	0.7180	0.031*	
H2D	0.1052	−0.1306	0.6391	0.031*	
O1B	0.03022 (13)	0.20461 (10)	0.97277 (8)	0.0357 (2)	
H1B	−0.0805	0.2095	0.9912	0.054*	
O2B	−0.28171 (13)	0.17175 (11)	0.95706 (8)	0.0424 (3)	
O3B	−0.31379 (13)	0.06993 (12)	0.85167 (10)	0.0492 (3)	
Cl1A	0.70902 (4)	0.37097 (3)	0.13787 (3)	0.03519 (10)	
Cl1B	0.18542 (4)	0.12266 (3)	0.36875 (2)	0.03163 (10)	

### Atomic displacement parameters (Å^2^)

**Table:**

	*U*^11^	*U*^22^	*U*^33^	*U*^12^	*U*^13^	*U*^23^
C1A	0.0284 (6)	0.0254 (6)	0.0266 (7)	−0.0009 (5)	−0.0051 (5)	−0.0051 (5)
C2A	0.0210 (6)	0.0246 (6)	0.0276 (7)	−0.0023 (5)	−0.0007 (5)	−0.0024 (5)
C3A	0.0258 (6)	0.0239 (6)	0.0309 (7)	−0.0044 (5)	−0.0065 (5)	−0.0039 (5)
C4A	0.0286 (6)	0.0240 (6)	0.0247 (6)	−0.0008 (5)	−0.0042 (5)	−0.0041 (5)
C5A	0.0213 (6)	0.0327 (7)	0.0350 (7)	−0.0018 (5)	−0.0002 (5)	−0.0087 (6)
C6A	0.0238 (6)	0.0334 (7)	0.0394 (8)	−0.0037 (5)	−0.0074 (6)	−0.0119 (6)
C7A	0.0336 (7)	0.0331 (7)	0.0283 (7)	0.0017 (6)	−0.0063 (6)	−0.0088 (6)
C8A	0.0502 (9)	0.0437 (8)	0.0341 (8)	−0.0092 (7)	−0.0013 (7)	−0.0208 (7)
N1A	0.0250 (6)	0.0310 (6)	0.0382 (7)	−0.0029 (5)	0.0015 (5)	−0.0055 (5)
N2A	0.0277 (5)	0.0285 (5)	0.0241 (5)	−0.0061 (4)	−0.0002 (4)	−0.0083 (4)
O1A	0.0370 (5)	0.0461 (6)	0.0373 (6)	−0.0035 (5)	−0.0040 (5)	−0.0213 (5)
O2A	0.0373 (6)	0.0484 (6)	0.0471 (7)	−0.0046 (5)	0.0121 (5)	−0.0213 (5)
O3A	0.0232 (5)	0.0635 (7)	0.0721 (8)	−0.0122 (5)	0.0004 (5)	−0.0258 (6)
C1B	0.0277 (6)	0.0275 (6)	0.0248 (6)	−0.0024 (5)	−0.0058 (5)	−0.0066 (5)
C2B	0.0207 (6)	0.0238 (6)	0.0269 (6)	−0.0030 (5)	−0.0032 (5)	−0.0045 (5)
C3B	0.0236 (6)	0.0245 (6)	0.0287 (7)	−0.0049 (5)	−0.0061 (5)	−0.0065 (5)
C4B	0.0244 (6)	0.0231 (6)	0.0251 (6)	−0.0025 (5)	−0.0046 (5)	−0.0044 (5)
C5B	0.0211 (6)	0.0338 (7)	0.0320 (7)	−0.0029 (5)	−0.0052 (5)	−0.0085 (6)
C6B	0.0258 (6)	0.0382 (7)	0.0353 (7)	−0.0063 (5)	−0.0100 (6)	−0.0128 (6)
C7B	0.0292 (6)	0.0286 (6)	0.0264 (7)	−0.0069 (5)	−0.0011 (5)	−0.0072 (5)
C8B	0.0434 (8)	0.0395 (8)	0.0332 (8)	−0.0029 (6)	0.0012 (6)	−0.0193 (6)
N1B	0.0233 (5)	0.0322 (6)	0.0359 (7)	−0.0050 (5)	−0.0010 (5)	−0.0098 (5)
N2B	0.0232 (5)	0.0282 (5)	0.0255 (5)	−0.0020 (4)	−0.0036 (4)	−0.0084 (4)
O1B	0.0335 (5)	0.0462 (6)	0.0327 (5)	−0.0062 (4)	−0.0038 (4)	−0.0200 (4)
O2B	0.0322 (5)	0.0554 (6)	0.0411 (6)	−0.0068 (5)	0.0061 (4)	−0.0237 (5)
O3B	0.0251 (5)	0.0671 (7)	0.0690 (8)	−0.0133 (5)	−0.0015 (5)	−0.0376 (6)
Cl1A	0.02968 (17)	0.0419 (2)	0.02772 (18)	−0.00866 (14)	−0.00213 (13)	−0.00206 (14)
Cl1B	0.02849 (17)	0.03788 (18)	0.02609 (18)	−0.00808 (13)	−0.00452 (13)	−0.00475 (13)

### Geometric parameters (Å, °)

**Table:**

C1A—O1A	1.3378 (15)	C1B—O1B	1.3413 (15)
C1A—C6A	1.3924 (18)	C1B—C6B	1.3925 (18)
C1A—C2A	1.3981 (18)	C1B—C2B	1.3996 (17)
C2A—C3A	1.3908 (18)	C2B—C3B	1.3882 (17)
C2A—N1A	1.4425 (16)	C2B—N1B	1.4473 (15)
C3A—C4A	1.3713 (18)	C3B—C4B	1.3755 (17)
C3A—H3A	0.9500	C3B—H3B	0.9500
C4A—C5A	1.4008 (18)	C4B—C5B	1.3994 (17)
C4A—C7A	1.5000 (18)	C4B—C7B	1.5030 (17)
C5A—C6A	1.3682 (19)	C5B—C6B	1.3691 (18)
C5A—H5A	0.9500	C5B—H5B	0.9500
C6A—H6A	0.9500	C6B—H6B	0.9500
C7A—N2A	1.4928 (16)	C7B—N2B	1.4852 (15)
C7A—H7A	0.9900	C7B—H7C	0.9900
C7A—H7B	0.9900	C7B—H7D	0.9900
C8A—N2A	1.4759 (16)	C8B—N2B	1.4788 (16)
C8A—H8A	0.9800	C8B—H8D	0.9800
C8A—H8B	0.9800	C8B—H8E	0.9800
C8A—H8C	0.9800	C8B—H8F	0.9800
N1A—O3A	1.2109 (15)	N1B—O3B	1.2125 (14)
N1A—O2A	1.2406 (15)	N1B—O2B	1.2350 (14)
N2A—H2A	0.9200	N2B—H2C	0.9200
N2A—H2B	0.9200	N2B—H2D	0.9200
O1A—H1A	0.8400	O1B—H1B	0.8400
			
O1A—C1A—C6A	116.81 (12)	O1B—C1B—C6B	117.37 (11)
O1A—C1A—C2A	126.21 (12)	O1B—C1B—C2B	125.88 (12)
C6A—C1A—C2A	116.98 (12)	C6B—C1B—C2B	116.74 (11)
C3A—C2A—C1A	121.68 (12)	C3B—C2B—C1B	122.28 (11)
C3A—C2A—N1A	117.71 (11)	C3B—C2B—N1B	117.56 (11)
C1A—C2A—N1A	120.61 (11)	C1B—C2B—N1B	120.15 (11)
C4A—C3A—C2A	120.34 (12)	C4B—C3B—C2B	119.98 (11)
C4A—C3A—H3A	119.8	C4B—C3B—H3B	120.0
C2A—C3A—H3A	119.8	C2B—C3B—H3B	120.0
C3A—C4A—C5A	118.47 (12)	C3B—C4B—C5B	118.22 (11)
C3A—C4A—C7A	119.74 (12)	C3B—C4B—C7B	122.67 (11)
C5A—C4A—C7A	121.76 (12)	C5B—C4B—C7B	119.08 (11)
C6A—C5A—C4A	121.07 (12)	C6B—C5B—C4B	121.63 (12)
C6A—C5A—H5A	119.5	C6B—C5B—H5B	119.2
C4A—C5A—H5A	119.5	C4B—C5B—H5B	119.2
C5A—C6A—C1A	121.45 (12)	C5B—C6B—C1B	121.13 (12)
C5A—C6A—H6A	119.3	C5B—C6B—H6B	119.4
C1A—C6A—H6A	119.3	C1B—C6B—H6B	119.4
N2A—C7A—C4A	111.77 (10)	N2B—C7B—C4B	113.02 (10)
N2A—C7A—H7A	109.3	N2B—C7B—H7C	109.0
C4A—C7A—H7A	109.3	C4B—C7B—H7C	109.0
N2A—C7A—H7B	109.3	N2B—C7B—H7D	109.0
C4A—C7A—H7B	109.3	C4B—C7B—H7D	109.0
H7A—C7A—H7B	107.9	H7C—C7B—H7D	107.8
N2A—C8A—H8A	109.5	N2B—C8B—H8D	109.5
N2A—C8A—H8B	109.5	N2B—C8B—H8E	109.5
H8A—C8A—H8B	109.5	H8D—C8B—H8E	109.5
N2A—C8A—H8C	109.5	N2B—C8B—H8F	109.5
H8A—C8A—H8C	109.5	H8D—C8B—H8F	109.5
H8B—C8A—H8C	109.5	H8E—C8B—H8F	109.5
O3A—N1A—O2A	122.35 (12)	O3B—N1B—O2B	122.23 (11)
O3A—N1A—C2A	119.40 (12)	O3B—N1B—C2B	118.92 (11)
O2A—N1A—C2A	118.25 (11)	O2B—N1B—C2B	118.84 (10)
C8A—N2A—C7A	112.24 (11)	C8B—N2B—C7B	111.57 (10)
C8A—N2A—H2A	109.2	C8B—N2B—H2C	109.3
C7A—N2A—H2A	109.2	C7B—N2B—H2C	109.3
C8A—N2A—H2B	109.2	C8B—N2B—H2D	109.3
C7A—N2A—H2B	109.2	C7B—N2B—H2D	109.3
H2A—N2A—H2B	107.9	H2C—N2B—H2D	108.0
C1A—O1A—H1A	109.5	C1B—O1B—H1B	109.5
			
O1A—C1A—C2A—C3A	179.66 (12)	O1B—C1B—C2B—C3B	179.51 (12)
C6A—C1A—C2A—C3A	−0.25 (19)	C6B—C1B—C2B—C3B	−1.17 (19)
O1A—C1A—C2A—N1A	0.0 (2)	O1B—C1B—C2B—N1B	0.5 (2)
C6A—C1A—C2A—N1A	−179.92 (11)	C6B—C1B—C2B—N1B	179.83 (11)
C1A—C2A—C3A—C4A	−0.80 (19)	C1B—C2B—C3B—C4B	0.92 (19)
N1A—C2A—C3A—C4A	178.87 (11)	N1B—C2B—C3B—C4B	179.94 (11)
C2A—C3A—C4A—C5A	1.30 (18)	C2B—C3B—C4B—C5B	0.13 (18)
C2A—C3A—C4A—C7A	−176.71 (11)	C2B—C3B—C4B—C7B	−178.01 (11)
C3A—C4A—C5A—C6A	−0.77 (19)	C3B—C4B—C5B—C6B	−0.89 (19)
C7A—C4A—C5A—C6A	177.19 (12)	C7B—C4B—C5B—C6B	177.32 (12)
C4A—C5A—C6A—C1A	−0.3 (2)	C4B—C5B—C6B—C1B	0.6 (2)
O1A—C1A—C6A—C5A	−179.13 (12)	O1B—C1B—C6B—C5B	179.78 (12)
C2A—C1A—C6A—C5A	0.8 (2)	C2B—C1B—C6B—C5B	0.4 (2)
C3A—C4A—C7A—N2A	−121.52 (13)	C3B—C4B—C7B—N2B	−46.81 (16)
C5A—C4A—C7A—N2A	60.54 (16)	C5B—C4B—C7B—N2B	135.07 (12)
C3A—C2A—N1A—O3A	4.67 (18)	C3B—C2B—N1B—O3B	3.65 (18)
C1A—C2A—N1A—O3A	−175.66 (12)	C1B—C2B—N1B—O3B	−177.30 (12)
C3A—C2A—N1A—O2A	−175.38 (12)	C3B—C2B—N1B—O2B	−176.18 (11)
C1A—C2A—N1A—O2A	4.30 (18)	C1B—C2B—N1B—O2B	2.86 (18)
C4A—C7A—N2A—C8A	173.76 (12)	C4B—C7B—N2B—C8B	−166.60 (11)

### Hydrogen-bond geometry (Å, °)

**Table:**

*D*—H···*A*	*D*—H	H···*A*	*D*···*A*	*D*—H···*A*
N2A—H2A···Cl1A	0.92	2.23	3.1301 (11)	167
N2A—H2B···Cl1B	0.92	2.18	3.0898 (11)	173
O1A—H1A···O2A	0.84	1.89	2.5917 (14)	140
O1A—H1A···Cl1B^i^	0.84	2.87	3.3918 (10)	122
N2B—H2C···Cl1A^ii^	0.92	2.17	3.0775 (11)	168
N2B—H2D···Cl1B^iii^	0.92	2.26	3.1671 (10)	168
O1B—H1B···O2B	0.84	1.88	2.5860 (14)	141

Symmetry codes: (i) −*x*, −*y*+1, −*z*+1; (ii) −*x*+1, −*y*, −*z*+1; (iii) −*x*, −*y*, −*z*+1.
